# Castalin Induces ROS Production, Leading to DNA Damage and Increasing the Activity of CHK1 Inhibitor in Cancer Cell Lines

**DOI:** 10.3390/antiox14091096

**Published:** 2025-09-08

**Authors:** Margherita D’Angelo, Annamaria Medugno, Maria Cuomo, Maria Carmen Ragosta, Andrea Russo, Giulio Mazzarotti, Giuseppe Maria Napolitano, Carmelina Antonella Iannuzzi, Francesco Errichiello, Luigi Frusciante, Martino Forino, Raffaele Cucciniello, Canio Martinelli, Annamaria Salvati, Domenico Memoli, Giovanni Nassa, Enrico Bucci, Michelino De Laurentiis, Antonio Giordano, Luigi Alfano

**Affiliations:** 1 Dipartimento di Salute Mentale, Fisica e Medicina Preventiva, Università degli Studi della Campania “Luigi Vanvitelli”, 80131 Napoli, Italy; margheritadangelo.dangelo@studenti.unicampania.it; 2 SHRO Italia Foundation ETS, 10060 Candiolo, Turin, Italy; a.medugno1@student.unisi.it (A.M.); m.cuomo@student.unisi.it (M.C.); m.ragosta@student.unisi.it (M.C.R.); a.russo@ssmeridionale.it (A.R.); g.mazzarotti@student.unisi.it (G.M.); 3 Department of Medical Biotechnologies, University of Siena, 53100 Siena, Italy; 4 Clinical and Translational Oncology Program, Scuola Superiore Meridionale (SSM, School of Advanced Studies), University of Naples Federico II, 80131 Napoli, Italy; g.napolitano@ssmeridionale.it; 5 Department of Breast and Thoracic Oncology, Istituto Nazionale Tumori-IRCCS-Fondazione G. Pascale, 80131 Napoli, Italy; c.iannuzzi@istitutotumori.na.it (C.A.I.); m.delaurentiis@istitutotumori.na.it (M.D.L.); 6 Department of Agricultural Sciences, Grape and Wine Science Division, University of Napoli Federico II, 83100 Avellino, Italy; francesco.errichiello@unina.it (F.E.); fruscian@unina.it (L.F.); forino@unina.it (M.F.); 7 Department of Chemistry and Biology ‘Adolfo Zambelli’, University of Salerno, 84084 Fisciano, Italy; rcucciniello@unisa.it; 8 Sbarro Institute for Cancer Research and Molecular Medicine, Center for Biotechnology, College of Science and Technology, Temple University, BioLife Science Bldg. Suite 333, 1900 N 12th Street, Philadelphia, PA 19122, USA; canio.martinelli@temple.edu (C.M.); enrico.bucci@temple.edu (E.B.); 9 Laboratory of Molecular Medicine and Genomics, Department of Medicine, Surgery and Dentistry “Scuola Medica Salernitana”, University of Salerno, 84132 Salerno, Italy; asalvati@unisa.it (A.S.); dmemoli@unisa.it (D.M.); gnassa@unisa.it (G.N.); 10 Molecular Pathology and Medical Genomics Unit, AOU “S. Giovanni di Dio e Ruggi d’Aragona”, University of Salerno, 84132 Salerno, Italy

**Keywords:** DNA damage response, DNA repair, CHK1 inhibitor, castalin, chestnut shell, natural extract

## Abstract

(1) Background: The use of cancer therapy is one of the most challenging arguments in cancer research and is in constant development. One of the principal problems connected with tumor therapy arises from the potential side effects connected with the classical chemotherapeutic treatment but also with molecular target therapy. The identification of novel molecules useful for the reduction of potential side effects but also as a new therapeutic opportunity is one of the hottest topics. (2) Methods: We identified castalin from chestnut shells by using NRM and LC-MS/MS. We treated different cancer cell lines with castalin alone or in combination with a CHK1 inhibitor. Finally, we performed an RNA-seq analysis of HeLa cells treated with castalin. (3) Results: We demonstrated the ability of castalin to induce DNA damage, probably by increasing ROS production. Consistently, antioxidant treatment, with ascorbic acid, reduced the DNA damage induced by castalin. Finally, we demonstrated the potential synergistic effect of castalin with SRA737, a CHK1 inhibitor currently used in clinical trials. (4) Conclusions: We demonstrated the ability of castalin to induce DNA damage favoring NHEJ repair. Moreover, the use of castalin in combination with SRA737 increased the efficacy of the CHK1 inhibitor, reducing its possible side effects.

## 1. Introduction

DNA damage is one of the leading causes of genome instability and serves as a predisposing factor for cancer development and progression [1]. Certain DNA damage response (DDR) proteins are implicated in cancer-prone syndromes, such as Ataxia Telangiectasia (ATM) and Nijmegen Breakage Syndrome (Nibrin), linking them to tumor development. Conversely, DNA repair defects represent an “Achilles’ heel” of cancer, as they make cancer cells more susceptible to treatments involving DNA-damaging agents, such as radiation therapy or cisplatin. These therapies induce various types of DNA lesions (e.g., single- or double-strand breaks), although side effects on healthy tissues limit their clinical use [2]. In this context, the identification of novel therapeutic strategies based on individual genetic makeup could be essential for improving cancer treatment. Many sources of DNA damage, both endogenous and environmental, have been shown to increase the production of reactive oxygen species (ROS). Chemotherapeutic agents like doxorubicin and cisplatin elevate ROS levels and exploit this increase as part of their mechanism of action [3]. Various naturally derived molecules have demonstrated antioxidant properties [4], as well as the ability to modulate the DNA damage response (DDR) [5,6]. ROS activate DDR pathways through sensor kinases such as ATM and ATR, which regulate Chk1 and Chk2 proteins [7,8] impacting on cell cycle progression and DNA repair. Excessive ROS levels can destabilize the DNA double helix and induce nucleotide alterations, leading to the accumulation of DNA damage and impaired DNA replication [9]. ROS can oxidize purines and pyrimidines, which are primarily repaired by base excision repair (BER) mechanisms [10]. However, when ROS cause more extensive DNA damage, nucleotide excision repair (NER) is the first line of response [11]. Additionally, ROS can indirectly induce double-strand breaks (DSBs) by generating single-strand breaks (SSBs) during DNA replication [12].

Currently, the balance between the pro-oxidant and antioxidant roles of ROS in tumorigenesis remains a topic of intense investigation. As mentioned, the antioxidant activity of the heme oxygenase 1 gene (HMOX1) plays a significant role in cancer development by reducing oxidative stress within cancer cells [13]. On the other hand, certain oncogenes increase ROS production, which contributes to tumor initiation, progression, and metastasis [14]. Cancer therapies, including conventional chemotherapy and molecular-targeted treatments, modulate ROS levels, influencing therapeutic responses and possibly contributing to variability in patient outcomes. Many FDA-approved cancer drugs function by upregulating ROS levels to promote cancer cell death and prevent drug resistance [14]. Moreover, ROS can modulate the activity of multidrug resistance (MDR) proteins by affecting mitochondrial metabolism and reducing ATP production, which is essential for MDR function. This suggests a potential role for ROS in enhancing the efficacy of chemotherapeutic drugs [15].

In our study, we aimed at identifying novel bioactive molecules from natural extracts that could enhance cancer therapy by increasing its efficacy or reducing associated side effects [5,6]. Here, we report the identification of **c**astalin [16], an ellagitannin found in chestnut shells, which induces DNA damage in cancer cell lines through ROS production, resulting in cytotoxic effects.

## 2. Materials and Methods

### 2.1. Cell Culture

Cervix adenocarcinoma (HeLa), human triple-negative breast cancer (TNBC) model (MDA-MB-231), and pleural effusion of hormone-dependent human breast carcinoma (MCF-7) cells were obtained from the American Type Culture Collection (ATCC). HeLa and MDA-MB 231 were cultured in Roswell Park Memorial Institute (RPMI) 1640 medium (Thermo Fisher Scientific, Waltham, MA, USA). An MCF-7 cell line was cultured in Dulbecco’s Modified Eagle Medium (DMEM) (Thermo Fisher Scientific, Waltham, MA, USA). All media were supplemented with 10% fetal bovine serum (FBS) (Thermo Fisher Scientific), 100 U/mL penicillin, 100 µg/mL streptomycin, and 2 mM L-glutamine. Cells were maintained at 37 °C in a humidified incubator with 5% CO_2_. Castalin was purchased from Phytolab (Vestenbergsgreuth, Germany)

### 2.2. Western Blotting

For total protein extraction we used a lysis buffer as described [5]. Lysates were clarified by centrifugation at 10,000× *g* for 20 min at 4 °C, and total protein concentration was determined using the Bradford assay (Bio-Rad, Hercules, CA, USA). Equal amounts of protein (50 μg per sample) from control and treated cell lysates were loaded onto 6–10% SDS–polyacrylamide denaturing gels. After electrophoretic separation, proteins were transferred to nitrocellulose (NC) membranes. Non-specific binding was blocked using 5% (*w*/*v*) non-fat dry milk (PanReac AppliChem, A0830, Darmstadt, Germany) dissolved in Tris-buffered saline with 0.1% (*v*/*v*) Tween-20 (TBS-T). Membranes were incubated overnight at 4 °C with the following primary antibodies, diluted in blocking buffer: anti-Lamin A/C (1:1000, #4777, Cell Signaling Technology, Danvers, MA, USA), anti-phospho-CHK1 (Ser345) (1:1000, #2348, Cell Signaling Technology), anti-CHK1 (1:1000, #H2714, Santa Cruz Biotechnology, Dallas, Texas, USA), and anti-GAPDH (1:1000, #2118, Cell Signaling Technology). Chemiluminescent signals were acquired using the Invitrogen™ iBright™ CL1500 Imaging System. The phosphorylated proteins, analyzed using Fiji software (version 2.14.0/1.54f), were normalized to the values of the corresponding total proteins, which were themselves normalized to the protein loading control.

### 2.3. Immunofluorescence Analysis

HeLa cells were fixed with 4% paraformaldehyde and permeabilized with 0.2% Triton X-100 followed by blocking with 1% BSA for 10 min at room temperature (RT). Cells were then incubated for one hour with the following primary antibodies: anti-53BP1 (1:500, NB100-304, Novus Biologicals, Centennial, Colorado, USA), anti-RIF1 (1:300, A300-567A, Bethyl Laboratories, Montgomery, Texas, USA), anti-phospho-DNA-PKcs (Ser2056) (1:200, ab124918, Abcam, Cambridge, UK), anti-γH2AX Ser139 (1:200, ab2893, Abcam, Cambridge, UK), and anti-γH2AX Ser139 (1:200, 05-636, Millipore, Burlington, MA, USA). AlexaFluor 647 donkey anti-mouse IgG (H + L), AlexaFluor 647 donkey anti-rabbit IgG (H + L), AlexaFluor 488 goat anti-mouse IgG (H + L), and AlexaFluor 488 goat anti-rabbit IgG (H + L) (all from Thermo Fisher Scientific) were used as secondary antibodies. Coverslips were mounted onto glass slides using ProLong™ Gold Antifade Mountant with DAPI (Thermo Fisher Scientific). We acquired the immunofluorescence images with confocal Zeiss LSM 900 Airyscan2 with a 63×/1.4 NA objective (Zeiss, Oberkochen, Germany). All the images were processed with Zen 3.9 software (Zeiss). Fluorescence foci per cell were quantified using Fiji software (version 2.14.0/1.54f).

### 2.4. Cell Proliferation Assay

HeLa, MCF-7, and MDA-MB 231 cells were seeded in triplicate into 96-well plates at densities of 1500, 3500, and 3000 cells per well, respectively, and allowed to adhere for 24 h. Upon 72 h of incubation, cells were fixed with 10% (*v*/*v*) trichloroacetic acid and stained with 0.4% (*w*/*v*) sulforhodamine B (SRB) in 1% (*v*/*v*) acetic acid. Cell viability was expressed as a percentage relative to untreated control cells, which were set at 100%. Data are presented as the mean ± standard deviation (SD) of three independent experiments (*n* = 3). We used one-way ANOVA, or Student *t*-test, for statistical analysis followed by multiple comparison post-tests to assess differences between groups. The castalin IC50 was determined by a dose–response curve using GraphPad Prism 10 Software.

### 2.5. Natural Comet Assay

HeLa cells were pre-treated for 3 h with castalin at concentrations of 62 µg/mL or 124 µg/mL, or with DMSO as a control. A total of 6 × 10^4^ cells were resuspended in 0.6% low-melting-point agarose (Sigma-Aldrich, St. Louis, MO, USA) and mounted on glass microscope slides. The slides were then incubated for 1 h at 4 °C in lysis buffer containing 2.5 M NaCl, 0.1 M EDTA, 10 mM Tris-HCl (pH 10), and 0.5% Triton X-100. Following lysis, slides were washed three times with electrophoresis buffer (Tris/Acetic Acid/EDTA) and subjected to electrophoresis at 0.6 V/cm for 30 min at room temperature. Slides were then rinsed with 1X PBS and stained with SYBR Green (Thermo Fisher Scientific) for 15 min at room temperature. After staining, slides were washed twice with 1X PBS and once with 70% ethanol, then air-dried overnight at room temperature. Comet tail analysis was carried out using OpenComet software (version 1.3.1).

### 2.6. Detection of Radical Oxygene Species

We detected the ROS levels by using 2′,7′-dichlorodihydrofluorescein diacetate (DCFH-DA) (US Biological Life Sciences, Salem, Mariland, USA HeLa cells (1.5 × 10^5^) were seeded into 35 mm dishes with 28 mm bottom wells and cultured for 24 h. After incubation, cells were washed twice with phenol red-free RPMI and treated for 3 h with castalin (124 µg/mL), hydrogen peroxide (400 µM), or DMSO at 37 °C in a 5% CO_2_ atmosphere. Following treatment, cells were incubated with 50 µM DCFH-DA for 30 min at 37 °C under 5% CO_2_. After incubation, cells were washed again, fixed with 4% paraformaldehyde, and stained with Hoechst to visualize nuclei.

### 2.7. Mitotic Catastrophe

HeLa cells were treated with indicated drug concentrations for 6 h followed by fixing with 4% paraformaldehyde. Cells were then permeabilized with 0.2% Triton X-100, and blocked with 1% BSA for 10 min at RT. Cover glass slides were incubated ON at 4 °C with the following primary antibody: anti-Phospho-Histone H3 (Ser10) (D2C8, Alexa Fluor 488 Conjugate) (1:200, #3465, Cell Signaling Technology). The following day, coverslips were mounted onto glass slides using ProLong™ Gold Antifade Mountant with DAPI (Thermo Fisher Scientific).

### 2.8. Reagents for NMR

Aqueous solutions were prepared with Milli-Q water from Millipore (Burlington, MA, USA). Deuterated solvents for NMR-based analyses were purchased from Cambridge Isotope Laboratories, Inc. (Tewksbury, MA, USA).

### 2.9. Chestnut Shell Collection, Extraction, and Chromatographic Separation

Chestnut shells (*Castanea sativa*) were kindly supplied by a company operating in Avellino, Italy. The obtained biomass was dried at 40 °C, ground, and stored at 4 °C before use. Successively, the ground material was extracted twice overnight with an ethanol–water solution (8:2). The extracts were concentrated under vacuum and partitioned twice against ethyl acetate. The acetate fraction was dried and partly used for pharmacological tests, partly for chemical investigation.

### 2.10. NMR and LC-MS/MS Analyses of the Ethyl Acetate Chestnut Shell Extract

1^H^ (600 MHz) NMR spectra were measured on a Bruker spectrometer. Chemical shifts were referenced to the residual solvent signal (CD3OD: δH 3.31, δC 49.3 ppm). LC-MS/MS analyses were performed on an Agilent Ultivo Triple Quadrupole coupled with an HPLC Agilent 1260 infinity II LC apparatus (Santa Clara, CA, USA). Chromatographic separation was obtained on a TosoH TSK-GEL column (250 × 2 mm) (Sigma-Aldrich, Milan, Italy) eluted with a 30 min linear gradient from 90% of B to 50% B (Buffer A: H2O 0.1% Formic Acid and Buffer B: Acetonitrile 0.1% Formic Acid) with a flow of 0.3 mL/min. The ESI source was operated in negative ion mode. The acquisition range was m/z 100–1400. Quantitative analyses were conducted by using the following standards: ellagic acid (Sigma-Aldrich, Milan, Italy) and gallic acid (Sigma-Aldrich, Milan, Italy). Linear regression analysis was performed using the Analyst 1.6.2 Software provided by the manufacturer (AB Sciex, Milan, Italy). Measurements were performed in triplicate. Due to the absence of standards, castalin isomers, corilagin isomers, and castalagin isomers were quantified by assuming the same molar response as ellagic acid; di-, tri-, tetra-, and penta-galloyl glucose isomers were quantified by assuming they had the same molar response as gallic acid.

### 2.11. NMR Data of Ellagic Acid

NMR data for ellagic acid were obtained by using CD_3_OD as a deuterated solvent at 25 °C (600 MHz): C1/C1′ 112.8; C2/C2′ 136.3; C3/C3′ 139.7; C4/C4′ 148.1; C5/C5′ 110.3; C6/C6′ 108.1; C7/C7′ 160.1; H5/H5′ 7.56 (singlet).

### 2.12. RNA-Seq and Analysis

RNA extraction, sample QC, library preparations, and sequencing reactions were conducted at GENEWIZ, LLC/Azenta. (South Plainfield, NJ, USA). Total RNA was extracted from frozen cell samples using a Qiagen RNeasy Plus Universal mini kit (Qiagen, Hilden, Germany) and RNA samples were quantified using a Qubit 3.0 Fluorometer (ThermoFisher Scientific, Waltham, MA, USA). The integrity of RNA was checked with the 4200 TapeStation (Agilent Technologies, Palo Alto, CA, USA). The preparation of RNA sequencing libraries was carried out by using the NEBNext Ultra II RNA Library Prep Kit for Illumina using the manufacturer’s instructions (New England Biolabs, Ipswich, MA, USA). Briefly, mRNAs were initially enriched with Oligod (T) beads. Enriched mRNAs were fragmented for 15 min at 94 °C. First-strand and second-strand cDNA were subsequently synthesized. cDNA fragments were end repaired and adenylated at 3′ends, and universal adapters were ligated to cDNA fragments, followed by index addition and library enrichment by PCR with limited cycles. The sequencing library was validated on the Agilent TapeStation (Agilent Technologies, Palo Alto, CA, USA), and quantified by using a Qubit 3.0 Fluorometer (ThermoFisher Scientific, Waltham, MA, USA) as well as by quantitative PCR (KAPA Biosystems, Wilmington, MA, USA). Sequencing libraries were multiplexed and clustered on an Illumina NovaSeq flow cell according to the manufacturer’s instructions. Samples were sequenced using a 2 × 150 bp paired-end (PE) configuration. Image analysis and base calling were performed by NovaSeq Control Software (NCS, Version v1.8). Raw.bcl files were converted to FASTQ and demultiplexed using bcl2fastq v2.20, then subjected to quality control with FASTQC (https://www.bioinformatics.babraham.ac.uk/projects/fastqc/(accessed on 25 June 2025)) and MULTIQC [17]. The adapter removal was assessed using Cutadapt [18]. Then, filtered reads were aligned to the human genome (hg38 assembly) considering genes annotated in GenCode Release 40 using STAR v2.7.11b with default parameters [19]. After gene expression quantification by featureCounts [20], differentially expressed genes were identified using DESeq2 [21], fixing cut-off expression at least ≥10 raw reads, while the differential expression was defined as fold-change (FC) ≥ |1.5|, with the corresponding adjusted *p*-value ≤ 0.05 calculated according to the Benjamini–Hochberg procedure. Functional analysis of differentially expressed transcripts was conducted using Ingenuity Pathway Analysis (IPA, QIAGEN) [22]. RNA-Seq data are avaible https://identifiers.org/arrayexpress:E-MTAB-15334 (accessed on 25 June 2025).

### 2.13. Statistical Analysis

Statistical analyses were performed using GraphPad Prism v9 for Mac. Differences between two groups were assessed with a two-sided Student’s *t*-test, while differences among multiple groups were evaluated using one-way ANOVA. *p* < 0.05 was considered statistically significant. The number of independent experiments and exact *p*-values are reported in the figure legends. For RNA-seq data, statistical analyses were conducted using R (v4.0.2). For each statistical analysis, we applied the Welch correction in cases of heteroscedasticity; moreover, the statistical tests employed were those commonly used for comparable analyses between different samples.

## 3. Results

### 3.1. Castalin Induces Cell Death in Cancer Cell Models

**Figure 1:**
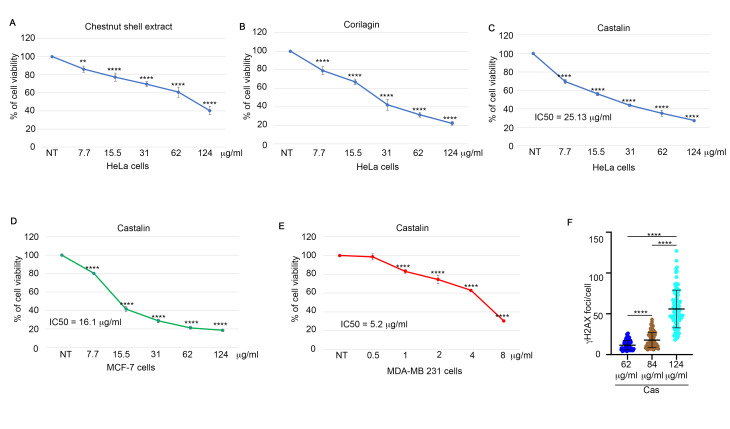
**Castalin affects cell viability of cancer cells.** (**A**) HeLa cells were treated with the indicated doses of chestnut shell extract and incubated for 72 h followed by cell viability assay. Results are presented as the mean ± standard deviation (SD) from three independent experiments. Statistically significant differences are indicated as ** *p* < 0.01 and **** *p* < 0.0001. (**B**) A cell viability assay on HeLa cells incubated for 72 h with indicated doses of corilagin. Data are presented as the mean ± SD from three independent experiments **** *p* < 0.0001. (**C**) Castalin-treated cells, with the indicated doses, for 72 h followed by SRB analysis. Statistically significant differences are indicated by **** *p* < 0.0001. The results are expressed as the mean ± SD from three independent experiments. (**D**) MCF-7 cells were treated with castalin for 72 h followed by a cell viability assay. Here we represent the mean ± SD from three experiments. **** *p* < 0.0001. (**E**) MDA-MB 231 triple-negative breast cancer cells were treated with different doses of castalin followed by a cell viability assay. We performed three independent experiments followed by representation of the mean ± SD. **** *p* < 0.0001. (**F**) HeLa cells were incubated with different doses of castalin followed by three hours of incubation. γH2AX foci/cell were analysed by using Fiji software. Thirty cells were analyzed for each condition. The results are presented as the mean ± SD from three independent experiments. Statistically significant differences are indicated by **** *p* < 0.0001. The statistical analyses were made with Welch’s-corrected one-way ANOVA. Welch’s correction was applied to avoid distortion due to the heteroscedasticity of the examined sample.

Our aim was to identify potential new molecules for cancer therapy by exploring different natural matrices, such as industrial waste [5]. In this study, we evaluated the extract from chestnut shells as a potential source of novel bioactive molecules. Specifically, the extract from chestnut shells was prepared as described in the Section 2, followed by solubilization and treatment of HeLa cells for 72 h, after which SRB analysis was performed. As shown in Figure 1A, the extract exhibited a dose-dependent cytotoxic effect on these cell lines. Given the extract’s ability to induce HeLa cell death, we proceeded to identify the active molecules present in this extract through NMR and LC-MS/MS analysis. A preliminary 1D-NMR analysis of the ethyl acetate extract, obtained as reported in Materials and Methods, was conducted with the purpose of identifying major occurring metabolites. The first metabolite that was straightforwardly identified was ellagic acid. Indeed, the ^1^H-NMR spectrum of the extract contained a diagnostic singlet resonating at d_H_ 7.56 ppm, typical for the equivalent protons at position 5 of ellagic acid. Moreover, in the ^13^C-NMR spectrum, we could identify all of the ellagic acid carbon signals whose chemical shifts were in full agreement with those reported in the literature [23]. Finally, the occurrence of ellagic acid was confirmed by means of mass spectrometry experiments acquired in negative ion mode that afforded a monocharged ion peak [M-H]^−^ at *m*/*z* 301.2 producing characteristic fragmentations at *m*/*z* 257 and 229 [24]. In addition to ellagic acid, the cross-interpretation of ^1^H- and ^13^C-NMR spectra allowed us to infer the presence of a complex mixture of ellagitannins (S1C and S1D). More specifically, resonances in the spectral region ranging from 3.7 to 5.5 ppm along with signals typical for anomeric protons around 6.6 ppm were indicative of glucose residues. Such a hypothesis was corroborated by carbon signals resonating across the region spanning from 65 ppm to 75 ppm along with anomeric carbon signals around 90 ppm. Additionally, proton resonances around 7 ppm together with carbon signals between 108 and 145 ppm were indicative of hydroxylated aromatic moieties. Finally, signals resonating between 160 and 170 ppm were attributed to ester functionalities. In order to tentatively identify specific ellagitannins present in the extract under investigation, we resorted to mass spectrometry. On the basis of the relevant elution order (retention times) and MS/MS data consistent with those available in the literature [25], along with gallic acid, some ellagitannins were tentatively identified as reported in Table S1.

In the extract we could identify the ellagitannins castalin **[16]** and corilagin **[26]** which are commonly found in various plants (Figure S1C,D). Thus, we tested these two tannins on HeLa cell viability and observed a similar trend in cytotoxicity (Figure 1B,C). In the scientific literature it is reported that corilagin is a potential anticancer agent, inducing G2/M checkpoint arrest in numerous cancer cell lines. Given the poorly characterized role of castalin in cell biology, we investigated its potential as an anticancer molecule. First, we tested the cytotoxic effect of castalin on other cancer cell models: breast adenocarcinoma (MCF-7) and triple-negative breast cancer (MDA-MB-231) cells. We observed consistent dose-dependent activity (Figure 1D,E), similar to the results obtained with HeLa cells. Overall, these data suggest that castalin was able to induce a cytotoxic effect in different cancer cell lines potentially useful for cancer therapy.

### 3.2. Castalin Activates the DNA Damage Response

**Figure 2:**
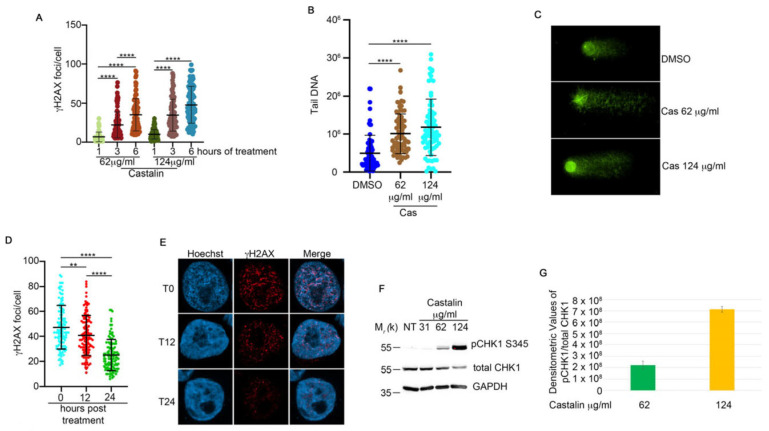
**Castalin induced DNA damage in HeLa cells.** (**A**) Castalin-treated cells at the indicated concentrations and incubated for three hours, followed by γH2AX immunofluorescence analysis. γH2AX foci were quantified using Fiji software. Thirty cells were analyzed for each condition. Results are presented as the mean ± SD from three independent experiments. Statistically significant differences are indicated as **** *p* < 0.0001. (**B**) A natural comet assay of HeLa cells treated for three hours with the indicated doses of castalin or DMSO. Comet analysis was performed using OpenComet software. Thirty cells were analyzed for each condition. Results are presented as the mean ± SD from three independent experiments. Statistically significant differences are indicated by **** *p* < 0.0001. Statistical analysis was carried out with Welch’s corrected one way ANOVA to avoid distortion due to the heteroscedasticity of the examined sample (**C**) HeLa comet images treated as in C. (**D**) We incubated HeLa cells with 124 μg/mL castalin for three hours, followed by drug washout to monitor γH2AX foci recovery as a readout of DNA repair. γH2AX foci were quantified using Fiji software. Results are presented as the mean ± SD from three independent experiments. Statistically significant differences are indicated as ** *p* < 0.01 and **** *p* < 0.0001. Thirty cells were analyzed per condition. Statistical analysis was performed by using the PRISM9 software** (version 10)** with Welch’s-corrected one-way ANOVA. Welch’s correction was applied to avoid distortion due to the heteroscedasticity of the examined sample. (**E**) Representative images from the experiment described in D. (**F**) HeLa cells treated with different doses of castalin for three hours were analyzed by Western blot using the indicated antibodies. (**G**) Densitometric analysis of pCHK1 S345 from HeLa cells treated with different doses of castalin.

To evaluate whether the cytotoxic effect of castalin on HeLa cells could be mediated by DNA damage, we treated the cells with different doses of castalin for three hours, followed by monitoring the activation of γH2AX as a marker of DNA damage [27]. Surprisingly, immunofluorescence analysis revealed a dose-dependent induction of DNA damage (Figure 1F and Figure S1A). Consistently, castalin also induced DNA damage in a time-dependent manner (Figure 2A and Figure S1B). The natural comet assay further confirmed a significant, dose-dependent DNA damage response upon castalin treatment (Figure 2B,C). To assess whether the induced DNA damage could be reversed by washout of the ellagitannin, we treated HeLa cells with 124 μg/mL of castalin for three hours, followed by replacement with a molecule-free medium to monitor the recovery of γH2AX foci. As shown in Figure 2D,E, castalin washout resulted in a time-dependent reduction in γH2AX foci.

**Figure 3:**
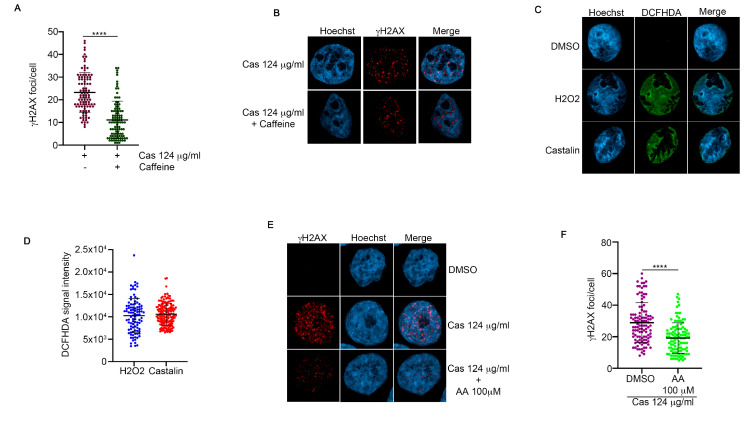
**Castalin leads to the induction of Radical Oxygen Species production.** (**A**) HeLa cells pre-treated with caffeine at 1mM, or DMSO, for one hour followed by incubation with γH2AX antibody. We analyzed thirty cells for each condition, from three independent experiments reporting the SD. **** *p* < 0.001. We used Welch’s-corrected **Student** *T-Test* for the statistical analysis to avoid distortion due to the heteroscedasticity of the examined sample (**B**) Representative images of HeLa cells, treated as in A. (**C**) Immunofluorescence images from HeLa cells treated with castalin at 124 µg/mL, H2O2 at 400 µM, or DMSO, followed by incubation for three hours. DCFH-DA staining was performed as described in the Material and Methods Section 2. (**D**) Statistical analysis of DCFH-DA signal intensity, measured by Fiji software, in HeLa cells treated as in E. Thirty cells were analyzed for each condition. Results are presented as the mean ± SD from three independent experiments. We used Welch’s-corrected Student *T-test* for the statistical analysis to minimize distortions caused by the heteroscedasticity of the sample under investigation (**E**) Representative images of HeLa cells, pre-treated with ascorbic acid (AA), at indicated concentrations for one hour or vehicle, followed by incubation with castalin for three hours. (**F**) Statistical analysis of HeLa cells treated as in A; γH2AX foci were analyzed by Fiji software. We monitored thirty cells for each condition, from three independent experiments reporting the SD. **** *p* < 0.001. The statistical analysis was carried out with Welch’s-corrected Student *T-test,* to reduce the effect of heteroscedasticity-related distortions in the analyzed sample.

To monitor if castalin was able to induce DDR activation, we examined one of the key checkpoint proteins, Checkpoint Kinase 1 (CHK1) [28]. To this end, HeLa cells were treated with different doses of castalin for three hours, followed by Western blotting for phospho-CHK1 (Ser345) [29], which revealed an increase in the activation of both proteins upon castalin treatment (Figure 2F,G). Given the critical roles of the apical protein kinases ATM and ATR in regulating DDR, we assessed the activation of γH2AX foci in the presence of castalin alone or in combination with caffeine, an inhibitor of both ATM and ATR kinases [30]. Analysis of γH2AX foci showed reduced foci formation when castalin was combined with caffeine, suggesting that the induced DNA damage signaling is dependent on ATM and ATR activity (Figure 3A,B). These data demonstrate that castalin induces DDR activation, inducing the activation of CHK1.

### 3.3. Castalin Was Able to Produce ROS Inducing the Activation of NHEJ

Many natural compounds, such as tannins, regulate the oxidative balance of cells, protecting membranes from lipid peroxidation and DNA damage [31]. To investigate whether castalin could be involved in modulating ROS production, we treated HeLa cells with the indicated amounts of castalin for three hours, followed by incubation with diacetyl-dichlorofluorescein (DCFH-DA), which is oxidized in the presence of ROS to produce the highly fluorescent DCF [32]. As shown by the fluorescence analysis reported in Figure 3C,D, castalin increases DCF fluorescence in a manner similar to H_2_O_2_, a well-known ROS producer in cells [33]. To demonstrate the direct involvement of ROS in the activation of γH2AX upon castalin treatment, we pre-treated HeLa cells with ascorbic acid (AA), an anti-ROS agent [34], followed by incubation with castalin. As shown in Figure 3E,F, the use of AA significantly reduced DNA damage, as measured by immunofluorescence γH2AX foci.

**Figure 4:**
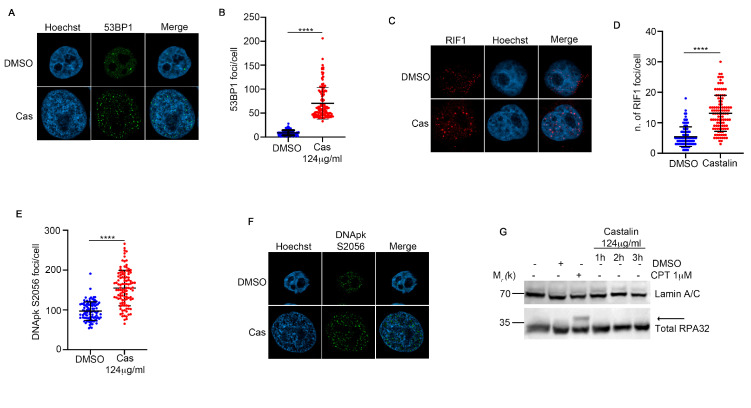
**Castalin treatment induces DNA damage leading to the activation of NHEJ.** (**A**) Images from 53BP1 immunofluorescence of castalin-treated cells for three hours. Hoechst was used as a nuclear marker. The castalin (Cas) image was obtained from the same sample used in Figure 2E (T0), which was double-stained to assess colocalization (**B**) 53BP1 foci analysis from HeLa cells treated with 124 µg/mL of castalin, for three hours, followed by Fiji software. Thirty cells were analyzed for each condition. Results are presented as the mean ± SD from three independent experiments. **** *p* < 0.0001. We used Welch’s-corrected Student *T*-Test for the statistical analysis to avoid inaccuracies resulting from the heteroscedasticity of the examined sample. (**C**) Illustration of RIF1 immunofluorescence of cells treated as in D. (**D**) RIF1 foci analysis with Fiji software from HeLa cells treated with castalin. Results are presented as the mean ± SD from three independent experiments. Statistically significant differences are indicated as **** *p* < 0.0001. The *Student T-test* with Welch’s correction was performed for the statistical analysis to prevent bias arising from the heteroscedasticity of the analyzed sample (**E**) DNA-PK S2056 foci/cell analysis with Fiji software from HeLa cells treated with the indicated dose of castalin and incubated for three hours. We monitored thirty cells for each condition representing the mean ± SD from three independent experiments The statistical analysis was carried out with the Welch’s-corrected *Student T-test *to avoid distortion due to the heteroscedasticity of the examined sample. Statistically significant differences are indicated as **** *p* < 0.0001 (**F**) Images from the immunofluorescence of HeLa wt, treated with castalin followed by DNA-PK S2056 staining. (**G**) Western blot analysis of HeLa cells treated with indicated doses of Castalin, DMSO or CPT followed by incubation with total RPA32 and Lamin A/C as DNA damage and protein loading controls, respectively.

HeLa cells treated with castalin exhibited double-strand breaks (DSBs), measured by γH2AX and comet assays (Figure 2). The two principal pathways involved in DSB repair are Non-Homologous End Joining (NHEJ) and Homologous Recombination (HR) [35]. The key difference between these repair mechanisms lies in their cell cycle distribution, with HR occurring exclusively during the S-phase, while NHEJ operates throughout the entire cell cycle. First of all, we monitored the immunofluorescence signal of p53-binding protein 1 (53BP1) and replication timing regulatory factor 1 (RIF1), two key players in NHEJ (Figure 4A–D) [36]. We demonstrated that castalin induced an increase in the number of 53BP1 and RIF1 foci. Consistently, we observed an elevated signal for DNA-PK at S2056, which is essential for promoting kinase dissociation from DNA and facilitating DNA end ligation, crucial for the proper completion of NHEJ (Figure 4E,F) [37]. Double-strand breaks are one of the most toxic lesions for cell viability and two principal mechanisms are deputated for their repair: NHEJ and homologous recombination (HR) [38]. Here, we treated HeLa cells with 124 μg/mL of castalin for indicated time points followed by Western blot analysis of total RPA32 protein. RPA32 is one of the most principal players of HR, in particular for DNA end resection. As shown in Figure 4G, castalin was not able to induce the appearance of the phosphorylated band of RPA32, which is activated in HR [39], with respect to camptothecin (CPT), which notoriously activated HR [40]. Overall, these data demonstrate that castalin induces ROS production, leading to the activation of NHEJ to repair DNA damage.

### 3.4. Castalin Potentiates the Effect of a CHK1 Inhibitor

**Figure 5:**
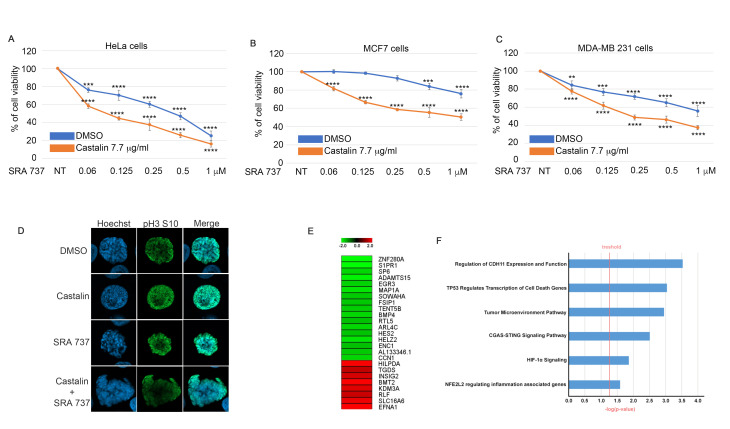
**Castalin was able to potentiate the effect of a CHK1 inhibitor on different cancer cell lines.** (**A**) Cell viability assay of HeLa cells treated with indicated doses of SRA737 with DMSO or castalin at 7.7 μg/mL for 72 h. *** *p* < 0.001 and **** *p* < 0.0001. Results are presented as the mean ± SD from three independent experiments. (**B**) MCF-7 cells were treated with SRA737 alone or in combination with castalin followed by the sulforhodamine B assay. Results are presented as the mean ± SD from three independent experiments. Statistically significant differences are indicated as *** *p* < 0.001 and **** *p* < 0.0001. (**C**) Combination treatment between a CHK1 inhibitor and castalin in MDA-MB 231 cells incubated for 72 h followed by cell viability assay. ** *p* < 0.01, *** *p* < 0.001 and **** *p* < 0.0001 The results are expressed as the mean ± standard deviation (SD) from three independent experiments. (**D**) HeLa cells incubated with castalin alone or in combination with SRA737 followed by immunofluorescence with the phospho H3 S10 antibody, as a mitotic marker, and stained with Hoechst as a DNA marker. (**E**) Heat map showing the top deregulated genes (padj ≤ 0.05) from RNA-seq data of HeLa cells treated with DMSO or castalin (3 μg/mL) for 72 h. (**F**) Bar chart generated through Ingenuity Pathway Analysis (IPA) functional annotation; depiction of statistically significant pathways enriched after castalin treatment.

Cancer therapy is continually evolving, particularly in the identification of novel protein targets. Recently, the CHK1 protein kinase has been under investigation in clinical trials as a new target for tumor treatment, both as monotherapy and in combination therapies [41]. We previously demonstrated that castalin activates CHK1 (Figure 2F,G). To further explore this, we examined the potential effect of combining castalin with the CHK1 inhibitor SRA737 [42] in inducing cancer cell death. First, we performed a Western blot analysis to monitor the phosphorylation of CHK1 at Ser345 upon treatment with castalin alone or in combination with SRA737. As reported in Figure S2A,B, both castalin and SRA737 alone induced phosphorylation of CHK1. Surprisingly, co-treatment with these compounds upregulated CHK1 S345 phosphorylation up to four times compared to treatment with either molecule alone (Supplementary Figure S2A,B). Next, to assess whether the combination of castalin and SRA737 could enhance the cytotoxic effect mediated by the CHK1 inhibitor, we performed a cell viability assay with different doses of SRA737, both alone and in combination with 7.7 μg/mL of castalin, across HeLa, MCF-7, and MDA-MB 231 cell lines (Figure 5A–C). We decided to use a dose of 7.7 μg/mL of castalin, for combination experiments, in order to test the effect of low doses of castalin on the activity of SRS737 reducing the possible off-targets phenomenon.

The results revealed increased toxicity with the combination treatment in all tested cancer cell lines. Given that castalin induces the phosphorylation of CHK1 as a checkpoint control, we next examined whether combining castalin with SRA737 could induce a checkpoint bypass, leading to the presence of unrepaired DNA and mitotic catastrophe [43]. To investigate this, we performed immunofluorescence analysis of HeLa cells treated with either molecule alone or with the combination of castalin and SRA737. As shown in Figure 5D, the combination treatment induced alterations in the nuclear compartment consistent with mitotic catastrophe. These results highlight the important role of castalin in enhancing the efficacy of the CHK1 inhibitor across different cancer cell lines, likely by inducing mitotic catastrophe. In order to demonstrate the possible involvement of castalin in the regulation of the RNA transcriptional program, we treated HeLa cells with **31 μg/mL** of castalin for three hours followed by RNA-seq analysis. Our analysis highlighted a total of 116 differentially expressed genes (Figure S2C, Table S2), and in Figure 5E, we report the top downregulated and upregulated mRNA identified in our screening. The single-gene analysis supports the role of castalin in HR downregulation and ROS generation. In particular, we observed a downregulation of ZNF280A, a positive regulator of the DNA end resection process [44]. Consistently, the downregulation of S1PR1 further supports the role of castalin as a ROS inducer, given S1PR1′s known involvement in the suppression of ROS generation [45]. Finally, we detected an upregulation of KDM3A, a histone demethylase [46], in castalin-treated cells. Recently, KDM3A was reported to be involved in cell cycle regulation upon DNA damage, promoting the alternative splicing of SAT1 mRNA [47], supporting a potential synergy between castalin and the cell cycle checkpoint inhibitor SRA737. Moreover, a functional annotation analyses obtained by Ingenuity Pathway Analysis (IPA) demonstrated the regulation of important pathways for cancer biology upon castalin treatment as the hypoxia and the TP53 transcriptional program as well as cell death pathways (Figure 5F). Overall, these data showed an important role of castalin in potentiating the effect of the CHK1 protein inhibitor underlying a possible translational relevance for cancer therapy.

## 4. Discussion

The identification of new molecules for tumor therapy is one of the hot topics in cancer research, given the emerging resistance mechanisms to both classical chemotherapy and novel targeted therapies [38]. One of the principal sources of molecules useful for cancer therapy has been natural extracts of diverse origins [48]. We previously identified oleanolic acid in fermented red grape pomace, a by-product of the oenological industry, which was able to enhance the efficacy of camptothecin treatment in cancer cell lines [49]. Here, we describe the potential use of castalin, derived from chestnut shells, as a DNA-damaging agent through the production of ROS, followed by γH2AX foci formation. This is one of the first reports describing a role for castalin in DNA damage. We identified this ellagitannin from the chestnut extract by NMR and LC-MS/MS analyses, which also revealed the presence of corilagin—another molecule belonging to the same chemical class but with a different role in cell cycle regulation, favoring arrest in the G2/M phase [50]. We demonstrated the activation of the NHEJ pathway in response to castalin, by indicating its involvement in DNA repair. NHEJ is one of the main mechanisms for repairing double-strand breaks, but it is error-prone, suggesting that the cytotoxic effect of castalin could also be mediated by low-fidelity DNA repair, increasing errors in the DNA sequence. An interesting finding was the activation of the CHK1 protein in response to castalin, in HeLa cells, depending probably on the ATR protein signaling which is reported to respond to ROS production [**51]**. CHK1 is a key checkpoint kinase involved in the DNA damage response, essential for cell cycle arrest to facilitate correct repair **[52]**. Emerging evidence has shown the potential efficacy of the CHK1 inhibitor SRA737 in cancer therapy, suggesting its use in combination treatments [42]. Given castalin’s role in activating CHK1 upon S345 phosphorylation, we hypothesized that SRA737 could be used to bypass the DNA damage checkpoint, leading to unrepaired DNA and ultimately mitotic catastrophe [43]. The combination of SRA737 and castalin increased cytotoxicity compared to SRA737 alone in HeLa, MCF-7, and MDA-MB-231 cells. We decided to use a dose of 7.7 μg/mL of castalin, for combination experiments, to test the effects of low castalin concentrations on the activity of SRS737, aiming to reduce possible off-target effects caused by higher doses. In clinical trials, SRA737 has generally induced mild diarrhea, nausea, and vomiting, but also dose-limiting toxicities such as gastrointestinal events, neutropenia, and thrombocytopenia [42]. We suggest that combining SRA737 with castalin could reduce these side effects by allowing a lower dose of SRA737 without compromising cytotoxic efficacy. Consistently, all these hypotheses regarding the potential translational relevance of castalin require further investigation in **in vivo **models to better define the possible clinical application of this ellagitannin. The potential use of castalin in combination with other DNA-damaging agents, such as olaparib or camptothecin, will be considered, opening new perspectives for cancer therapy. In particular, the combination with camptothecin depends on castalin’s ability to regulate the cell cycle. We demonstrated that castalin induces activation of NHEJ, which occurs throughout all phases of the cell cycle, but we did not observe activation of RPA32, a marker of HR. These data suggest that castalin may induce DNA damage and cell cycle arrest prior to the S-phase. Given that camptothecin induces DNA damage exclusively in S-phase cells, the combination of castalin and CPT warrants further investigation. Finally, we performed RNA-seq analysis of HeLa cells treated with castalin to assess the impact of the treatment on the cellular transcriptional program. This analysis confirmed castalin’s mechanism of action via ROS generation, demonstrated by the upregulation of HIF-1α signaling (Figure 5F), a key pathway activated in response to ROS [53]. Moreover, RNA-seq revealed other important pathways regulated by castalin, such as those related to the tumor microenvironment. This is a significant point to investigate, considering the emerging view of cancer as a systemic disease involving not only tumor cells but also complex interactions with non-cancer cells, which are present in the TME, in the context of cancer therapies [54]. We performed RNA-seq analysis exclusively in HeLa cells to investigate the impact of castalin on DNA damage, using one of the most widely employed cellular models for the characterization of DNA repair mechanisms. The results obtained from RNA sequencing in the other two breast cancer models could differ due to their genetic characteristics, such as the BRCAness phenotype of MDA-MB-231 cells. This hypothesis is further supported by the differential effects of castalin observed in cell viability assays, which showed varying responses across the tested cancer cell models. Finally, we assessed castalin activity only in cancer cell models, without evaluating its potential effects in non-cancerous cells. This represents an important aspect for future investigation. We speculate that castalin may be more effective in highly proliferative cells, potentially affecting the cell cycle and replication by ROS activity [55].

## Data Availability

For additional information or to request resources and reagents, please contact the lead author, Luigi Alfano, who will provide assistance (l.alfano@istitutotumori.na.it). Original Western blot images have been deposited at Zenodo at [https://zenodo.org/], doi 10.5281/zenodo.15738916 (accessed on 25 June 2025). Microscopy data reported in this paper will be shared by the lead contact upon request. RNA-seq raw sequencing data have been deposited in the ArrayExpress repository with the following accession numbers E-MTAB-15334.

## Supplementary Materials

The following supporting information can be downloaded at: https://www.mdpi.com/article/10.3390/antiox14091096/s1, Figure S1: (A) Representative images of HeLa cells treated with different concentrations of Castalin and incubated for three hours followed by immunofluorescence analysis of γH2AX foci. Hoechst as used as DNA staining marker. (B) HeLa cells were treated with different doses of Castalin and incubated for indicated time points. At the end of incubation time, we performed immunofluorescence analysis as in A. (C) Structure of ellagic acid and 1H NMR spectrum in CD3OD of the chestnut shell extract. (D) 13C NMR spectrum in CD3OD of the chestnut shell extract; Figure S2: (A) Western blot analysis of HeLa wt cells treated with Castalin alone or in combination with SRA737. GAPDH was used as protein loading control. (B) Densitometric analysis, by using Fiji software, of CHK1 phosphorylation at S345 from HeLa cells treated as in A. (C) Volcano plot depicting differentially expressed transcripts (FC |≤ 1.5|; adjusted *p*-value ≤ 0.05) between control (DMSO) and Castalin-treated (3 µg/mL) HeLa cells after 72 hours; Table S1: Major ellagitannins tentatively identified on the basis of their chromatographic and MS data. Quantifications are expressed as mg/mL of extract.
