# Supplementary material for: Castalin Induces ROS Production, Leading to DNA Damage and Increasing the Activity of CHK1 Inhibitor in Cancer Cell Lines
Source: Antioxidants (Basel). 2025 Sep 8;14(9):1096. doi: 10.3390/antiox14091096 (PMC12466859; doi:10.3390/antiox14091096)
Supplement: Supplementary file 1 [file antioxidants-14-01096-s001.zip › Table S1.pdf]

Table S1. Major ellagitannins tentatively identified on the basis of their chromatographic and MS data. Quantifications are expressed as mg/mL of extract.

Compound	retention time (min)	$[M-H]^-$	Fragments $[M-H]^-$	Concentration (μg/mL)
Ellagic acid	0.63	301.2	257; 229	1200.2 ± 21.1
Gallic Acid	0.75	169.1	125	280.4 ± 3.2
Digalloyl glucose	1.67	483.1	331; 271	167.7 ± 3.4
Trigalloyl glucose	1.98	635.1	483; 271	377.0 ± 4.1
Tetragalloyl glucose	2.34	787.1	635; 301	544.7 ± 4.4
Pentagalloyl glucose	3.43	939.1	787; 331	250.9 ± 3.8
Castalin isomer 1	11.86	630.9	483; 301	80.4 ± 3.2
Castalin isomer 2	13.74	631.2	483; 301	115.1 ± 4.6
Castalagin isomer 1	13.86	933.3	631; 301	104.5 ± 3.3
Castalagin isomer 2	13.96	933.5	631; 301	98.7 ± 3.4
Corilagin isomer	15.89	632.9	301	60.4 ± 4.1
Castalin isomer 3	17.51	631.0	483; 301; 275	200.2 ± 3.8
