# Supplementary material for: Castalin Induces ROS Production, Leading to DNA Damage and Increasing the Activity of CHK1 Inhibitor in Cancer Cell Lines
Source: Antioxidants (Basel). 2025 Sep 8;14(9):1096. doi: 10.3390/antiox14091096 (PMC12466859; doi:10.3390/antiox14091096)
Supplement: Supplementary file 1 [file antioxidants-14-01096-s001.zip › Supplementary Figure Legends.pdf]

Supplementary Figure S1. A) Representative images of HeLa cells treated with different concentrations of Castalin and incubated for three hours followed by immunofluorescence analysis of γH2AX foci. Hoechst as used as DNA staining marker. **B)** HeLa cells were treated with different doses of Castalin and incubated for indicated time points. At the end of incubation time, we performed immunofluorescence analysis as in A. **C)** Structure of ellagic acid and ^{1}H NMR spectrum in CD_3OD of the chestnut shell extract. **D)** ^{13}C NMR spectrum in CD_3OD of the chestnut shell extract.

Supplementary Figure S2. A) Western blot analysis of HeLa wt cells treated with Castalin alone or in combination with SRA737. GAPDH was used as protein loading control. **B)** Densitometric analysis, by using Fiji software, of CHK1 phosphorylation at S345 from HeLa cells treated as in A. **C)** Volcano plot depicting differentially expressed transcripts (FC $|\geq 1.5|$; adjusted p-value ≤ 0.05) between control (DMSO) and Castalin-treated (3 µg/mL) HeLa cells after 72 hours.
