# Supplementary figures and images for: Castalin Induces ROS Production, Leading to DNA Damage and Increasing the Activity of CHK1 Inhibitor in Cancer Cell Lines
Source: Antioxidants (Basel). 2025 Sep 8;14(9):1096. doi: 10.3390/antiox14091096 (PMC12466859; doi:10.3390/antiox14091096)

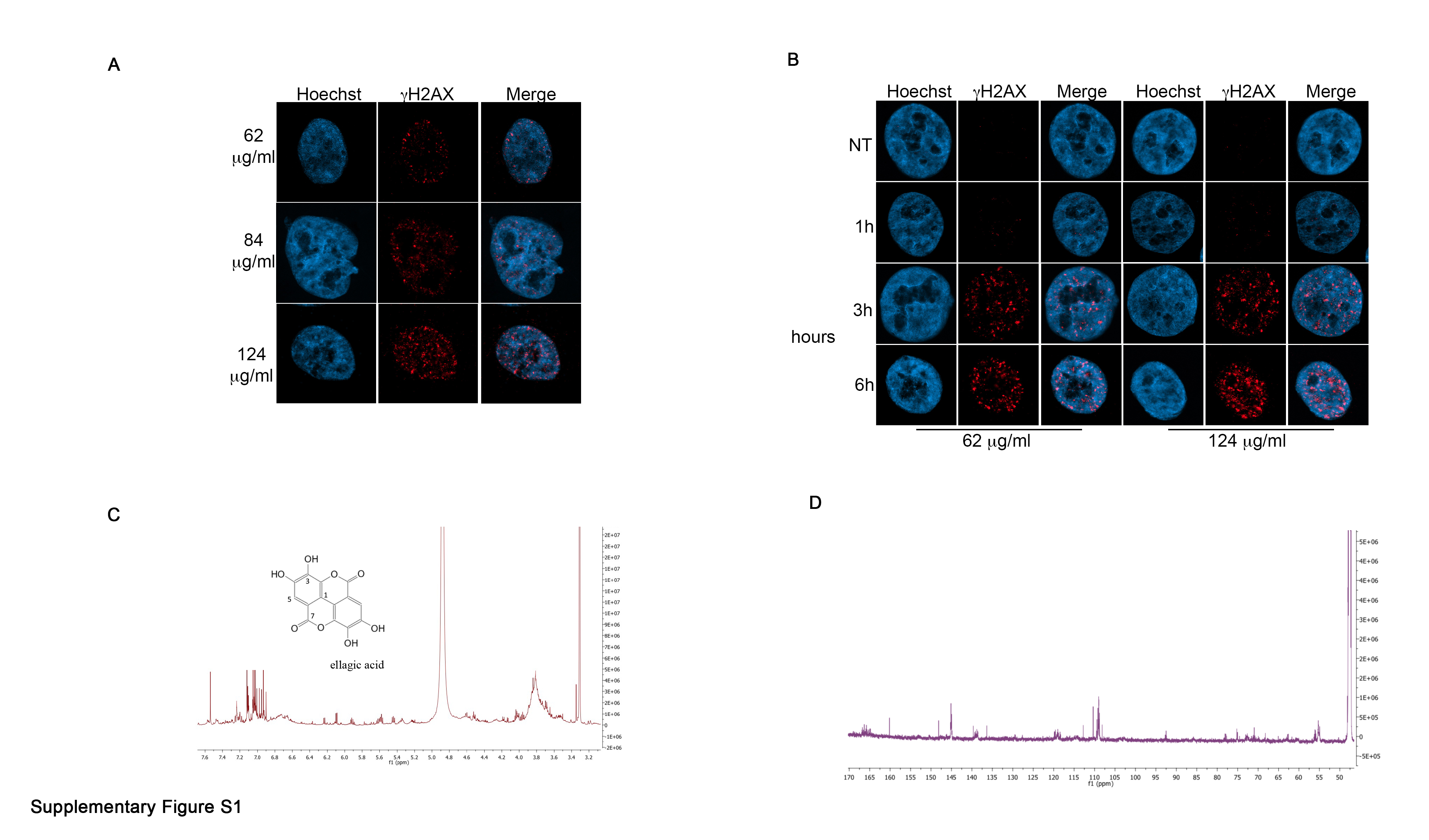

Supplement: Supplementary file 1 [file antioxidants-14-01096-s001.zip › Supplementary S1.jpg]

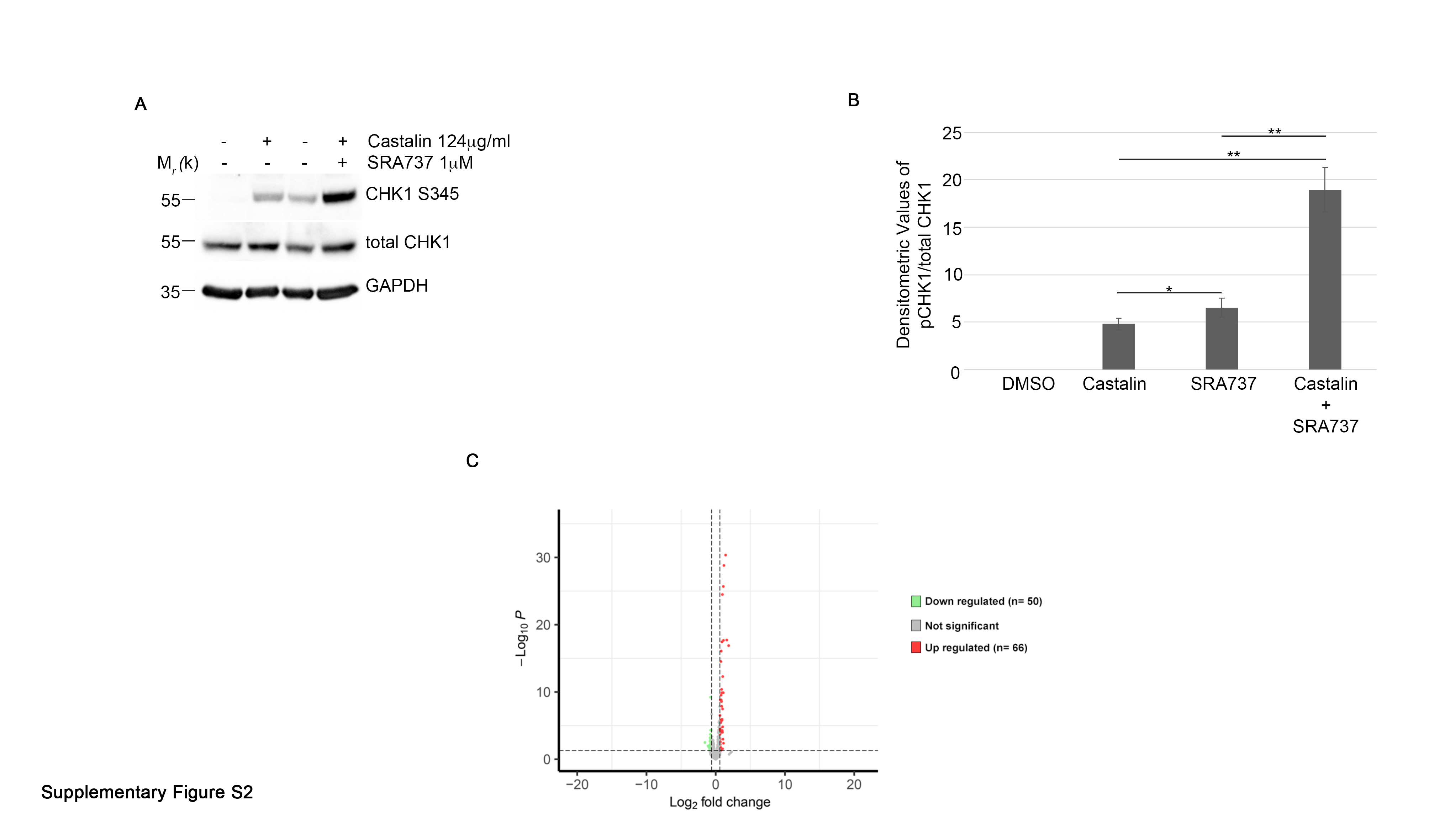

Supplement: Supplementary file 1 [file antioxidants-14-01096-s001.zip › Supplementary S2.jpg]
